# Robust capital cost optimization of generation and multitimescale storage requirements for a 100% renewable Australian electricity grid

**DOI:** 10.1093/pnasnexus/pgae127

**Published:** 2024-03-25

**Authors:** Raheel A Shaikh, David J Vowles, Alex Dinovitser, Andrew Allison, Derek Abbott

**Affiliations:** School of Electrical and Mechanical Engineering, The University of Adelaide, Adelaide, SA 5005, Australia; School of Electrical and Mechanical Engineering, The University of Adelaide, Adelaide, SA 5005, Australia; School of Electrical and Mechanical Engineering, The University of Adelaide, Adelaide, SA 5005, Australia; School of Electrical and Mechanical Engineering, The University of Adelaide, Adelaide, SA 5005, Australia; School of Electrical and Mechanical Engineering, The University of Adelaide, Adelaide, SA 5005, Australia

**Keywords:** Australian energy transition, long-duration energy storage, low-carbon systems, optimization for low-cost, optimal generation-storage mix

## Abstract

Transitioning from a fossil-fuel-dependent economy to one based on renewable energy requires significant investment and technological advancement. While wind and solar technologies provide lower cost electricity, enhanced energy storage and transmission infrastructure come at a cost for managing renewable intermittency. Energy storage systems vary in characteristics and costs, and future grids will incorporate multiple technologies, yet the optimal combination of storage technologies and the role of interconnectors in alleviating storage needs are not widely explored. This study focuses on optimal generation-storage capacity requirements to elucidate associated investments. We propose a multitimescale storage solution consisting of three storage categories and an interconnector between Australia’s eastern and western grids. Subsequently, through an extensive sensitivity analysis, we investigate the impact of specific storage technologies and cost variations. Our findings demonstrate that the proposed interconnector offers a cost-effective solution, reducing generation and storage power capacity needs by 6 and 14%, respectively, resulting in 4% savings on overall investment costs. Moreover, the study’s sensitivity analysis reveals that wind generation provides 50–70% of the energy demand for the least-cost solution. Despite storage inefficiencies, long-duration storage would need to be deployed to support power capacity for 2–4 days, representing 15–40% of peak demand, depending on future technology costs. Subsequently, achieving a fully renewable electricity sector in Australia requires a significant expansion of generation and storage infrastructure, with a 13-fold increase in storage power capacity and a 40-fold increase in storage energy capacity compared to existing levels.

**Significance Statement**

Australia is at the forefront of adapting renewable technologies and addressing policy concerns on technology selection. Our paper presents an optimization framework that uses real-world generation data to optimize the generation-storage mix for a minimum-cost solution. We consider utility solar, wind, and hydro for generation, while storage is characterized by duration and cost into shallow (<4 h), medium (4–16 h), and deep (>16 h) storage. The algorithm proposes optimum capacities for each technology required for a fully renewable grid. Additionally, we explore the impact of a new interconnector between the eastern and western Australian grids alongside existing transmission infrastructure and conduct sensitivity analyses on technology and cost.

## Introduction

Transitioning the energy sector toward low-carbon technologies such as solar, wind, and hydropower is essential for migrating from emissions-intensive fossil fuels. The power sector alone contributes to over one-third of the total global emissions annually (1); as such, the adoption of renewable sources in this sector can play a vital role in alleviating emissions from other sectors, such as transportation, industry, and agriculture. While fossil fuels provide 63% of the global electricity production presently (2), the share of renewables is expected to increase from the current 26.5 to 80% by 2050, with solar and wind power accounting for over half of total electricity generation (3).

The concept of the grid fully powered by renewables (RE100) has been explored extensively over the past decade (4–7). While some researchers have raised concerns about the technical feasibility of the RE100 grid, citing issues such as cost competitiveness, generation variability, storage inefficiencies, and resource limitations (8–10), recent research and developments in the renewable energy sector have addressed many of these criticisms (11–13). Despite the challenges involved, most studies agree on the feasibility of achieving near-zero emissions. Renewable energy adoption is multifaceted; besides the benefits of decarbonization, it also (i) alleviates a range of fossil-fuel-induced airborne pollutants and smog, (ii) creates energy independence, and hence potentially reduces geopolitical tensions, (iii) enables electrification and increased efficiencies through DC transmission (14), (iv) creates long-term sustainability (15), and (v) allows for rapid deployment compared to nuclear energy and mitigates the risks associated with nuclear proliferation (16). Consequently, renewable generation is expected to increase due to relatively lower production costs compared to fossil-fuel generation and the growing commitment of countries to achieve net-zero CO_2_(e) emissions by 2050 (17). Nevertheless, grid balancing is a significant technical challenge in integrating variable renewable energy (VRE) sources. The inherent intermittency and inflexibility of weather-dependent sources create imbalances between generation and demand. Therefore, it is crucial to implement solutions that provide the necessary dispatchability to ensure a cost-effective balance between supply and demand.

The introduction of energy storage systems (ESSs) is expected to contribute significantly to the solution of the demand–supply balancing problem caused by variability in power from VRE (18, 19), especially when renewable energy penetration in any grid exceeds 60–80% (7, 20–24). The required storage power capacity increases linearly with increasing VRE penetration, while the required energy capacity increases exponentially (25) for a given level of supply reliability (26). Moreover, the expansion of transmission networks can also assist in achieving decarbonization targets by reducing the need for ESS deployment and lowering investment costs (6, 27), allowing energy transfer over a large geographic area with relatively low losses, thus smoothing the variability of renewables.

Both transmission and storage provide flexibility to the power grid, with the former shifting supply spatially and the latter shifting it in time (temporally). However, the least-cost approach for achieving high VRE penetration remains unclear, and the amount of energy storage or transmission augmentation needed to ensure a stable VRE grid is not fully understood, as most studies in the literature differ in modeling assumptions and technologies utilized for supply–demand balancing. Accounting for diverse storage technologies with varying energy storage durations is crucial in identifying the most cost-effective way to achieve clean energy objectives.

This paper extends our previous research (28, 29) by conducting a more comprehensive examination of the optimal generation-storage mix needed to achieve 100% renewable electricity (RE100) in Australia, classifying the storage into three categories of shallow, medium, and deep storage based on duration (the ratio of energy capacity to power rating) and round-trip efficiency (RTE). The study incorporates several novel features: (i) a realistic representation of grid storage technologies based on expected cost and performance, contrasting with the single storage approach commonly used in previous studies; (ii) the utilization of high-resolution (30-min) real-world data from 116 existing generators across Australia, enabling the consideration of capacity factor variations across geographical regions; (iii) the investigation of the proposed interconnection between Australia’s eastern (National Electricity Market—NEM) and western (Wholesale Electricity Market—WEM) grids, including the existing interregional transmission links within the NEM; (iv) separate modeling of the NEM and WEM grids and analysis of the potential benefits of their integration; and (v) an extensive 2-fold sensitivity analysis, encompassing cost assumptions to understand the boundaries of the optimal solution while considering cost reductions and technology advancements.

## Literature review

Over the past decade, significant research has focused on renewable energy transition and decarbonization of energy systems. While most studies have examined the power sector, some interdisciplinary studies have employed intersector modeling approaches. These studies have varied in spatial resolution and utilized models with diverse generation, storage technologies, and transmission networks at various geographical scales, including countries (7, 30), continents, and the global scale (4, 5); authors in Breyer et al. (31) comprehensively review these studies. Nevertheless, variations in electricity consumption and generation patterns across countries necessitate individualized analyses for each case. Regional studies cannot fully represent the unique characteristics of other regions, emphasizing the importance of localized analyses to address specific contexts and requirements.

The study by Lu et al. (6) explores the renewable grid for South East Asia, with a focus on pumped hydro energy storage (PHES) and continental interconnectors using high voltage direct current (HVDC) technology. The study found that the interconnectors between regions reduced the required renewable generation capacity by 20% and the storage power and energy capacity requirements by 40 and 50%, respectively.

According to Schmidt et al. (32), various storage technologies can be utilized across the electricity supply chain. Energy storage is crucial in balancing supply and demand and maintaining grid stability through voltage, frequency, and inertia control. The authors analyze the levelized cost of storage for nine different technologies and various applications, including energy arbitrage and peaker plant replacement. They conclude that Li-ion batteries are economically viable for short to medium-duration storage (<8 h) to ensure grid stability. Hydrogen storage is more cost-effective than PHES and compressed air energy storage (CAES) for longer storage applications, ensuring energy adequacy. Nonetheless, the cost feasibility of existing electrochemical storage technologies, including Li-ion batteries, remains uncertain, with recent reports indicating increases in battery costs due to potential shortages of raw materials like lithium and nickel (33). Furthermore, to accommodate higher integration of VRE, dispatchable storage with increased flexibility is necessary—as demand side management (DSM) and virtual power plants (VPPs) are unable to balance the grid on their own (34).

Researchers have estimated the generation and storage need for different VRE penetrations in the Australian context. In Ref. (35), authors modeled a fully renewable grid with double the present-day demand due to the electrification of industry and transportation sectors and proposed off-river pumped hydro storage, indicating a need for 51 GW (10.5 h) of storage for Australian grids. Despite modeling several future demand scenarios, the study lacked detailed sensitivity analysis, only considered single utility storage, and proposed interconnector capacities that were unrealistically higher (roughly 16×) than existing capacities (5 GW).

Furthermore, previous attempts (22, 29, 36–38) to model a fully renewable Australian grid are noted. The authors in Ref. (36) proposed a low-cost grid with thermal storage integrated into concentrated solar thermal (CST). In Ref. (37), PHES with an optimum capacity of approximately 17 GW (26 h) was suggested for Australia. Conceptually similar to Ref. (36), authors in Ref. (38) proposed biofuel and CST technology as the dominant supplier in the generation mix. Nonetheless, these studies do not consider (i) diversity in the storage mix, (ii) utility-scale batteries, or (iii) long-duration storage; they also introduced rather optimistic low-cost assumptions. Similarly, in its latest plan (ISP 2022 (39)), the Australian energy market operator (AEMO) estimated storage with capacities of 45 GW (13 h) required by 2050 to meet a doubling in demand for a renewable penetration of 97%, with 30 GW of storage served by VPPs and vehicle-to-grid (V2G) storage, with 15 GW provided by utility-scale storage. However, AEMO’s study does not consider any interconnector between eastern and western grids, whereas a high-level study (40) estimated that interconnecting the two grids via HVDC would result in a cost reduction of A$1.9 billion, alleviating the need for 1 GW of generation and 500 MW of battery storage.

Several other studies modeling renewable grids have limitations; for example, (i) outdated or optimistic technology cost assumptions (6, 41) that call for reassessment due to rapid technology development, (ii) constraints on storage technologies by defining a specific energy-to-power ratio (38), and (iii) neglecting the benefit of the optimal mix of storage technologies and considering a single storage technology instead (37), such as batteries, CAES, or PHES with their respective round-trip efficiencies. In contrast, our work investigates optimal generation capacity requirements and the mix of multiple storage types based on various storage characteristics, i.e. cost, efficiency, duration, power, and energy capacities.

In light of the preceding considerations, this work employs an in-house mathematical model utilizing the latest data for Australian electricity grids to investigate cost-optimized long-term investment plans for achieving a 100% renewable energy grid. It examines the integration of renewable generation sources and storage technologies, providing insights into optimal allocations for solar and wind energy, required storage capacities, and potential cost reductions through regional interconnections. Sensitivity analysis explores the impact of future cost variations and technological developments on proposed requirements.

## Energy storage characterization for RE100

The energy storage technologies are designed to provide rated power for a certain duration, also known as the capacity energy-to-power ratio (${\mathrm{EP}}_{c}$). Long-term storage systems, such as those with large reservoirs, tend to have very high ${\mathrm{EP}}_{c}$ of several days to months. On the other hand, short-term storage systems, such as PHES with small reservoirs, tend to have an ${\mathrm{EP}}_{c}$ of only a few hours (42). While a grid partially powered by dispatchable fossil generators may need short-duration storage (<4 h), the dependency on storage increases rapidly as the renewable penetration rises over 90%, with storage needed for as much as 10–100 h (7). Therefore, we categorize storage, based on duration, into three categories: shallow (<4 h), medium (4–16 h), and deep (>16 h). While shallow storage is effective for providing grid services such as frequency and voltage control pertaining to their quick response time, medium and longer duration storage availability is essential to cover shortages that occur intraday, intraweek, intraseason, and interseason due to the weather-dependent nature of renewable generators, especially solar sources.

Grid-scale batteries, such as Li-ion and lead-acid, currently offer cost-effective storage of up to 4 h; however, as technology improves and costs decrease, longer storage durations over 6 h are expected to become economical. Nevertheless, integrating Li-ion batteries with wind or solar is not cost-effective beyond 6 h (34). Batteries provide quick response and high efficiency and are suitable for diurnal arbitrage. Grid-scale batteries such as the Victorian Big Battery (300 MW, 1.5 h) (43) and the Hornsdale Power Reserve (150 MW, 1.3 h) (44) were deployed due to their modularity and short installation time. On the other hand, PHES provides a cost-effective option for long-duration medium and deep storage (up to days) but is unsuitable for fast response applications and has an installation lead time of several years; it typically requires a large amount of space and is location-dependent. Also, it is subject to significant and time-consuming environmental approval processes; this is an important factor in consideration when the required timelines for grid transition to 100% renewable are now very short. Nevertheless, PHES is by far the most widely deployed technology, accounting for approximately 90% of global storage power (45); the technology has reached maturity and is unlikely to see cost reductions in the future.

Finally, power-to-gas-to-power, particularly with the use of green hydrogen (PtH_2_tP), offers the advantage of independent scaling of storage energy and power capacity and has the potential for extended energy storage over several weeks or seasons, despite lower overall RTE. Here, PtH_2_tP involves the production of green hydrogen gas through water electrolysis using renewable electricity (46), followed by storage and reelectrification via fuel cells or retrofitted combustion turbines. A recent fall in the cost of polymer exchange membrane (PEM) electrolyzers and fuel cell technology (47) has increased the potential of hydrogen as an alternative for heating and transportation fuel. Moreover, hydrogen can be shipped in compressed form or in the form of green ammonia to local and international markets (48). This spatial energy shift potentially results in cost reduction for power networks. The South Australian (SA) government has recently committed to a 250-MW hydrogen project near Whyalla, to be commissioned by 2025 (49).

Moreover, other storage technologies have the potential to provide for a range of durations, some of which include adiabatic CAES (50), liquid air energy storage (51), thermal storage, using molten salt or sand, and flow batteries (52). Due to distinct storage characteristics, the future grid is expected to host diverse storage technologies. Although an optimal mix of wind and solar PV exists for renewable electricity generation (53), the optimal mix will vary depending on the selection of storage technologies. Therefore, it is challenging to calculate the storage capacities needed and plan the investments required to transform the existing grid to RE100.

## Australian electricity market

**Fig. 1.**
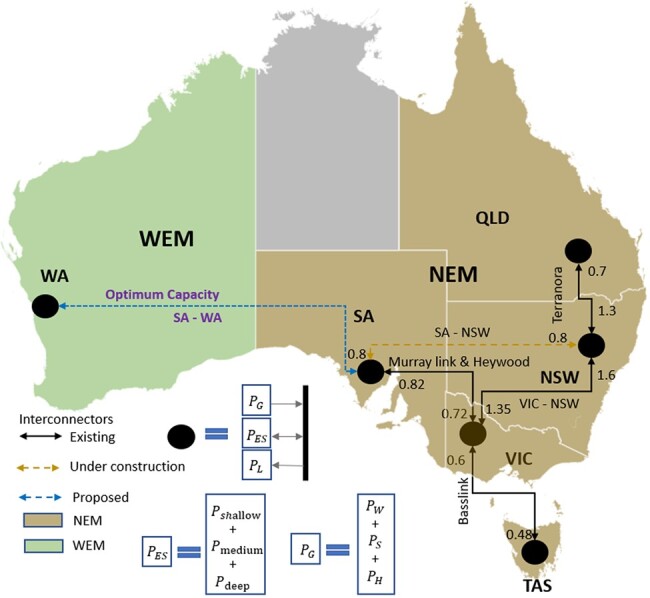
NEM interconnected with WEM, along with interconnectors between different regions of NEM with transmission line forward capacities in GW. The SA–NSW interconnector is under construction and included in our model. The capacity for multiple interconnectors connecting the same regions is aggregated to reflect simplified individual interconnections. The indicated regions are SA, QLD, NSW, VIC, WA, and TAS.

The Australian electricity sector comprises the NEM and other regional grids such as the WEM and the grid of the Northern Territory. The NEM is the largest interconnected grid in Australia, supplying electricity to SA, Tasmania (TAS), Victoria (VIC), New South Wales (NSW, including the Australian Capital Territory), and Queensland (QLD), while the WEM supplies electricity to the southwest of Western Australia (WA), refer to Fig. 1.

Australia’s electricity generation has traditionally relied heavily on fossil fuels; however, there has recently been a notable shift toward renewable energy sources. There has been a decline in the contribution of fossil fuel-based electricity over the past decade. Concurrently, VRE installation has grown significantly, increasing from 3 GW in 2010 to 28 GW in 2021. The remarkable expansion of VRE installations can be attributed primarily to solar photovoltaic (PV) systems, encompassing both rooftop and utility-scale installations. Solar PV has exhibited a compound annual growth rate (CAGR) of 33.2% over the decade, resulting in a 1,750% increase in capacity. Additionally, wind power capacities have experienced substantial growth, with a 480% increase over the same period and a CAGR of 17%.

With the increasing economic viability of renewable energy sources and the aging of conventional power generation capacity, the proportion of renewable energy sources in the Australian electricity market is expected to continue to increase to meet the objectives of reducing greenhouse gas emissions. In 2021, about one-third (32.5%) of Australia’s electrical energy was sourced from renewables, with the maximum instantaneous generation amounting to 64% for the NEM and 80% for the WEM (54). Energy storage technologies have also seen a decrease in costs, especially battery energy storage systems (BESS) (32).

The WEM is isolated from other grids and must balance the supply–demand with its own generation sources. As of 2021, the WEM is powered predominantly by fossil-fueled generators, with roughly 66% of annual energy from coal and gas, and the remaining from wind and solar generators. There are no hydroelectric generators to provide the WEM with dispatchable renewable electricity.

**Fig. 2.**
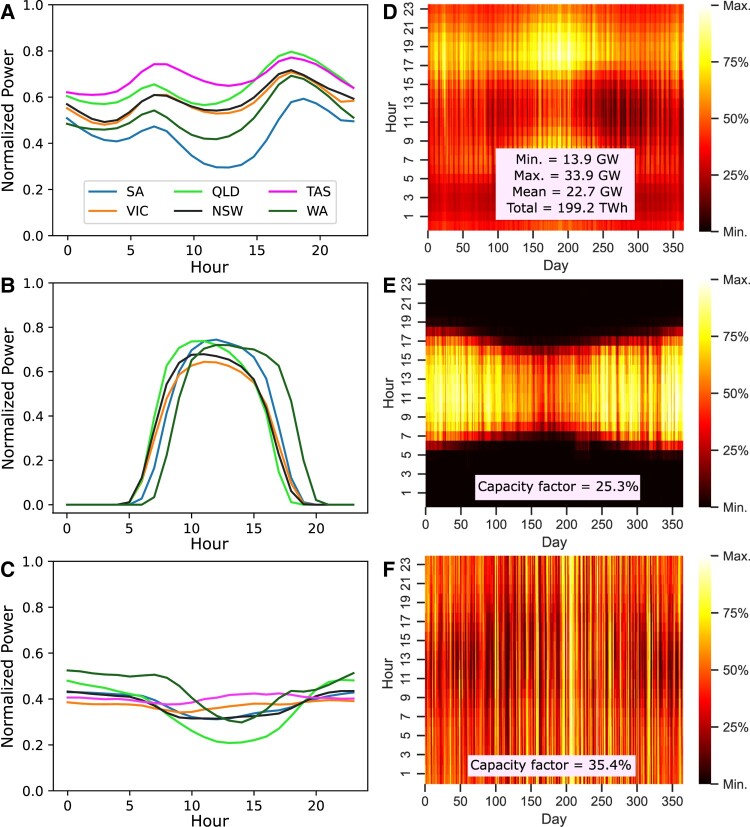
Daily mean demand a) and power generation from utility solar b) and onshore wind c) for each AEM region normalized to the maximum value. The actual combined load d) and generation profiles for utility solar e) and onshore wind f) for all hours and days of 2021 are also shown. The average annual capacity factor for solar and wind is 25.3 and 35.4%, respectively. Additionally, the mean demand for the AEM is 22.7 GW, with the maximum recorded at nearly 34 GW. The total grid demand experiences a minimum of only 14 GW at midday due to the offsetting contribution from rooftop-solar generation.

The normalized generation and load profiles for the combined NEM and WEM grids, referred to (in our study) as the Australian Electricity Market (AEM), are plotted in Fig. 2. We observe that solar and wind generation exhibit a weak negative correlation (r=−0.256) on an hourly scale. Hence, it can be inferred that they tend to complement each other throughout the day (55). Similarly, lower operational demand is observed during midday, especially in spring (Mar–May) and autumn (Sept–Nov), while the demand peaks in the evenings. There is a time difference of two hours between the eastern regions of the NEM and the WEM regions: Perth in WA is about 4,000 km west of Sydney. As such, while the sun sets in eastern regions, the solar panels in Western Australia (WA) may be able to provide for the evening peaks on the eastern seaboard, and similarly, though less important, the eastern states midmorning solar generation could provide for early morning peaks in WA. The interconnector, therefore, could provide an opportunity to manage the load and generation in an improved manner besides offering a path for exploiting all the solar and wind resources across the resulting energy corridor from SA to WA.

Our study utilizes actual generation data of 100 generators (comprising utility solar and onshore wind) in the NEM and 16 generators in the WEM, sampled at 30-min intervals for the year 2021. Additionally, we consider three storage options for each region: lithium-ion batteries (Li-ion), PHES, and power-to-gas-to-power (PtH_2_tP), chosen based on their suitability for short-term (shallow), medium-term (medium), and long-term (deep) storage, respectively. A total of 18 storage systems are assessed across six regions. The optimization objective is to minimize the overall system costs, which comprise investment costs of generation, various storage systems (considering power and energy capacity costs), along with the cost of new interconnections, subject to certain constraints. The optimization framework, the regional generation capacity, the individual generator information, including capacity factors, and the economic assumptions are outlined in the Supplementary material. The model is simulated under four scenarios, as listed in Table 1.

**Table 1.** Grid scenarios for least-cost optimization of generation and storage.

#	Scenario	Identifier	Definition
1	Copper plate^a^	CP	All regions, including WA, are assumed to be mutually interconnected with an ideal interconnector without any capacity constraints or losses.
2	Isolated grids	IG	This scenario is simulated to consider the possibility that all regions are disconnected from each other, and thus, individual regional requirements for generation and storage are calculated. Although unrealistic because the NEM is already (relatively weakly) interconnected, it provides insight into the benefits of reduced generation and storage resulting from interconnection.
3	Business-as-usual	BAU	This scenario is to simulate two independent grids, i.e. NEM with existing interconnectors and WEM in isolation from any other grid.
4	Australian Electricity Market	AEM	The last scenario is simulated to model the generation and storage needs should a new interconnector between NEM and WEM be constructed to combine the two grids.

^a^This is the idealization of the grid as a connection of all generators and loads by zero impedance conductors to a single node that provides a bound as a benchmark for comparison.

## Results

The simulation results for four interconnection scenarios as previously described in Table 1 are summarized in Table 2, while additional results are provided in the Supplementary material. The ideal copper plate (CP) scenario, i.e. ease of energy transfer across any region, leads to the lowest possible requirements for generation and storage. Conversely, the isolated grids (IG) scenario provides an upper bound on generation and storage capacities. As the level of interconnection increases, the proportion of wind energy in the energy mix also rises, reaching 66% in the copper plate scenario compared to 60% in the disconnected scenario. Furthermore, higher interregional connectivity reduces the optimal excess generation from 131% (IG) to 112% (CP) to achieve lower cost solutions. The IG scenario requires additional generation and storage power capacities of over 72 and 25 GW, respectively, which are substantially higher than the corresponding quantities of 54 GW (generation) and 16.5 GW (storage) for the CP scenario. The overall storage energy capacity of the CP scenario is less than one-quarter of that of the disconnected grid, amounting to 330 GWh. The IG scenario, with individual regions having self-sufficiency in terms of generation and storage, will need a combined capacity of over 1 TWh for deep storage (over 80 h) to meet all shortfalls when solar and wind cannot meet the required demand for multiple days.

**Table 2.** Electricity generation and storage for different scenarios and regions.

Grid	$\alpha_{\mathrm{opt}}$ ^a^	Wind (GW)	Solar (GW)	Shallow		Medium		Deep		Cost ($Bn)
				*P* (GW)	*E* (GWh)	*P* (GW)	*E* (GWh)	*P* (GW)	*E* (GWh)	
Copper plate (CP)										
AEM	1.12	32.8	21.4	4.43	10.8	2.05	11.5	10.06	308	108
Isolated grids (IG)										
NSW	1.27	17	8.43	2.51	7.02	1.29	11.54	3.69	172	52.5
QLD	1.30	8.27	16.0	0.07	0.27	5.06	43.6	2.89	339	52.2
VIC	1.28	6.97	4.6	1.17	2.94	0.79	4.67	3.20	285	30.4
SA	1.71	2.22	1.56	0.60	1.74	0.27	2.03	1.35	109	11.2
TAS	1.0	0.2	0.0	0.0	0.0	0.0	0.0	0.0	0.0	0.3
WA	1.46	3.78	3.28	1.05	2.49	0.45	4.17	1.54	124	17.0
AEM	1.3	38.4	33.9	5.4	14.5	7.9	66.0	12.7	1029	163
Business-as-usual (BAU)										
NEM	1.2	34.4	21.2	4.42	11.8	4.73	40.0	8.65	547	120
WEM	1.46	3.78	3.28	1.05	2.5	0.45	4.17	1.54	124	17.0
AEM	1.22	38.2	24.5	5.5	14.3	5.18	44.05	10.2	671	137
Australian Electricity Market (AEM)										
AEM	1.19	38.2	21.04	4.16	13.2	4.70	39.20	9.30	647	129.1

^a^The optimum over-capacity factor determined by the algorithm, that results in the least-cost generation-storage-interconnector solution.

The comparison between the business-as-usual (BAU) scenario and the connected Australian electricity market (AEM) scenario provides valuable insights. The BAU scenario requires additional generation and storage capacities of 62.6 GW (7 GW for WA) and 20.8 GW (3 GW for WA), respectively. In contrast, the AEM scenario requires over 59 and 18 GW of generation and storage power capacities, respectively, representing savings of 3.6 GW (6%) and 2.8 GW (14%) in power capacities with an optimal interconnector capacity of 1.7 GW connecting WA and SA. Additionally, the storage energy capacities reduce by around 29 GWh (4%) in AEM than BAU scenarios. The aggregate investments for the RE100 grid are expected to be under A$131.4 billion, with A$83 billion, A$46 billion, and A$2.3 billion allocated for generation, storage, and NEM-WEM interconnector, respectively. This technology mix represents an investment reduction of over A$5.3 billion (3.9%) compared with the current grid, where WEM is isolated from NEM (i.e. the BAU scenario). The additional generation and storage capacity requirements relative to the overall requirement are tabulated in Table 3. It is evident that deep storage is a significant component of the overall storage requirements, with power capacities accounting for over half of the overall power and energy capacities accounting for over 92% of the total storage energy. This suggests that deep storage is crucial for meeting the storage needs of the AEM grid.

**Table 3.** Additional capacities required relative to overall requirements for generation and storage in AEM (reference case).

Wind	Solar	Power capacity			Energy capacity		
		Shallow	Medium	Deep	Shallow	Medium	Deep
64.5%	35.5%	22.9%	25.8%	51.2%	1.9%	5.6%	92.5%

**Fig. 3.**
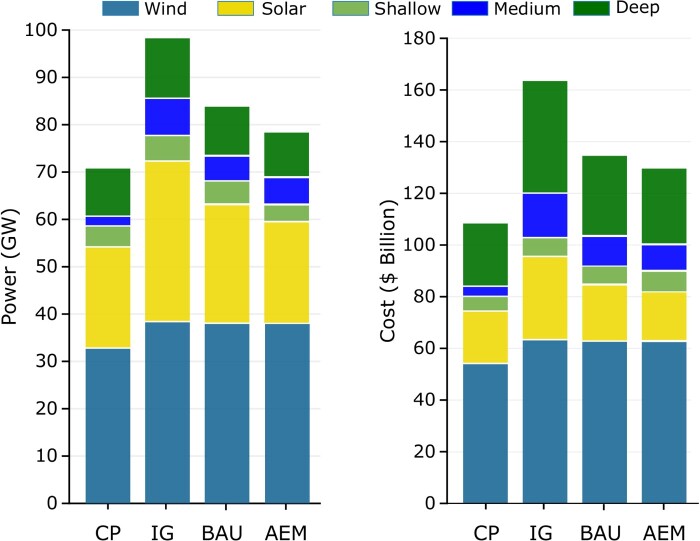
Accumulative power capacities for generation and storage power capacities in different scenarios. The CP and IG scenarios, though unrealistic, indicate the minimum and maximum capacities and costs. The AEM scenario has the lowest realistic investment cost, which is about A$5.3 billion less than the BAU scenario. After wind generation, the bulk of investment is required for deep storage.

The regionally required power capacities for each scenario are illustrated in Fig. 3. The NSW and QLD regions exhibit some of the highest power demands across all scenarios, primarily due to their higher energy consumption than other regions. Implementing interconnection between regions, from the IG to the AEM scenario, substantially reduces required power capacities. For instance, in the AEM scenario, the VIC region requires <9 GW, significantly lower than the 16.7 GW required for the IG scenario.

**Fig. 4.**
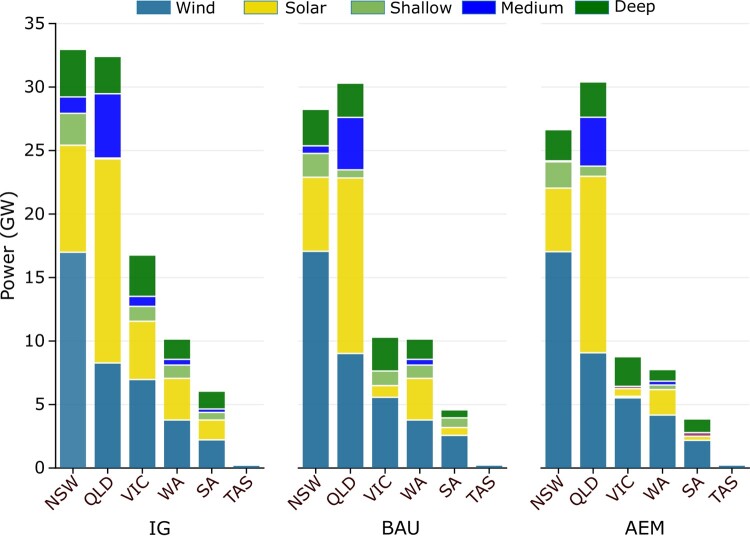
Power capacities required for each region under three scenarios. The NSW and QLD regions have the highest demand and therefore need over 25 GW of generation and storage capacities each. The TAS region does not need any storage due to the abundance of hydro generation capacity, while for TAS to have an RE100 grid, around 180 MW of wind needs to be installed.

Furthermore, introducing a new interconnector between WA and SA has a notable impact on the combined generation and storage power requirements. The power requirements reduce from 83.5 to 77.4 GW with an interconnector of 1.7 GW capacity. This reduction is due to energy transfer at peak power demands, which allows for time-shifting of energy. Overall, the interconnector results in a reduction of over 6 GW in the required capacities. Combined capacities in each scenario and the associated cost are plotted in Fig. 4.

### Storage requirements

**Fig. 5.**
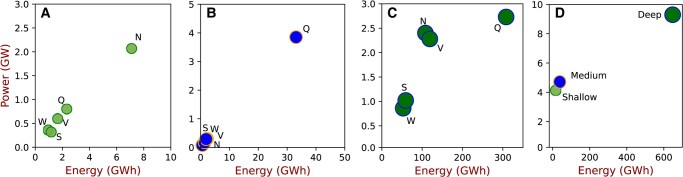
a) Shallow, b) medium, and c) deep storage requirements for each region in interconnected AEM scenario, while d) represents accumulative storage for AEM. Notations: T-TAS, V-VIC, S-SA, W-WA, N-NSW, and Q-QLD.

Upon examining the storage requirements based on duration and RTEs, it becomes apparent that even with high power cost and low efficiency associated with deep storage, the power capacity and energy capacity provided by deep storage exceeds 50 and 92%, respectively, of overall storage capacities. Fig. 5 shows the regional shallow, medium, and deep storage requirements for the AEM scenario. The QLD region has the highest medium and deep storage requirements compared to other regions, and while QLD has the second-highest demand of 53.4 TWh after NSW (66.2 TWh) in the modeled year, it has limited interconnected capacity, roughly 7.4% of peak demand (9.44 GW). The interconnector capacity to peak demand ratio for NSW and VIC is 27 and 34%, respectively. This underscores the importance of grid interconnection in shaping generation and energy storage requirements. Similarly, NSW has the highest shallow storage requirement with 2 GW/7 GWh. Other regions require <1 GW of shallow storage, providing 2–3 h at rated capacity. Deep storage requirements range from 61 h for WA (under 1 GW power capacity) to 113 h for QLD (2.7 GW power capacity). The TAS region does not need any storage due to extensive hydro resources and sufficient existing generation capacity.

The response or the state of charge (SOC) of shallow, medium, and deep storage in each region is examined (included in the Supplementary material). On average, deep storage has the highest average SOC, followed by medium and shallow storage; deep storage can maintain a high SOC due to its large energy capacity, enabling it to sustain longer periods of discharge before needing to recharge. On the other hand, medium and shallow storage have smaller energy capacities, resulting in more frequent charging and discharging cycles to maintain the required SOC. In absolute terms, the shallow storage for all regions exhibits over 200 cycles, meaning that it is fully discharged and charged over 200 times (with a depth of discharge [DOD] of 100% assumed for all storage in our simulations). However, the QLD shallow storage has 355 cycles, roughly one charge and discharge per day. This increased storage utilization is due to high solar penetration (over 55%) in QLD compared to all other regions. Alternatively, the deep storage has the lowest equivalent cycle ranging from 4 cycles annually for QLD to 17 for NSW and 16 for VIC. The high number of cycles for shallow storage in all regions indicates that it plays a crucial role in balancing the fluctuations in renewable generation and demand, while deep storage provides long-term energy storage and stability.

### Energy loss and curtailment

The primary purpose of the storage is to enable time-shifting of energy by charging when there is excess generation and discharging when there is a shortfall, and as such, in general, more storage results in a lower curtailment. The difference between the energy drawn from the grid to charge the storage and the energy supplied to the grid in the same period represents energy loss due to the inefficiency of the storage mechanism. Table 4 records the energy exchange by each regional storage unit, the corresponding losses resulting from charging and discharging processes, and curtailment for the AEM scenario. The findings reveal that the system suffers a loss of roughly 15.4 TWh due to inefficiencies, and over 19 TWh is curtailed, resulting in a total loss of 15% of the total generated energy. The comparison of the losses incurred through storage operations and curtailment for various scenarios is listed in Table 5. Notably, the interconnection between the eastern and western grids results in a 2% reduction in overall losses, equivalent to 7.5 TWh of energy.

**Table 4.** Energy exchange in TWh by regional storages for various scenarios and resultant operational energy loss (TWh) along with curtailment (TWh).

Region	Shallow		Medium		Deep		Curtailment
	Charge	Discharge	Charge	Discharge	Charge	Discharge	
NSW	2.5	2.2	0.1	0.1	6.4	3.1	6.5
QLD	1.0	0.9	10.3	8.3	7.5	3.8	5.9
VIC	0.6	0.6	0.3	0.2	6.4	3.1	3.3
WA	0.4	0.3	0.5	0.4	2.4	1.1	3.5
SA	0.4	0.3	0.4	0.3	2.3	1.1	0.2
AEM	4.83	4.36	11.56	9.29	24.92	12.26	19.34
Loss	0.47		2.3		12.67		

**Table 5.** Storage operational energy losses (TWh) and curtailment (TWh) in different scenarios, with total unutilized energy (in TWh and percentage of annual generation).

Scenario	Shallow	Medium	Deep	Curtailment	Total	Gen. (%)
CP	0.41	0.8	6.3	16.4	23.9	11
IG	0.45	3.3	13.6	43.4	60.8	23
BAU	0.50	2.5	12.4	26.9	42.3	17
AEM	0.47	2.3	12.7	19.3	34.8	15

**Fig. 6.**
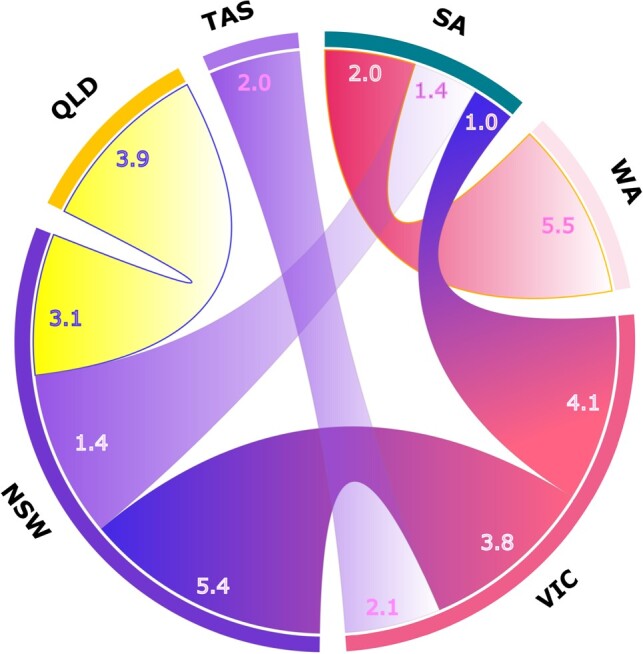
The representation of interconnectors between regions and the amount of energy exported in TWh. The value next to the region indicates the amount of energy exported to another region via the interconnection. The thickness of each chord also reflects the amount of exported energy.

The energy transported through interconnectors is explored, and the results are plotted in Fig. 6. The VIC and NSW regions are the largest net exporters in the simulations, each exporting around 10 TWh of energy through the three interconnectors. With the new interconnector between WA and SA, the WA region has a higher available generation and acts as a net exporter. Lastly, we model AEM with 82% renewable penetration to reflect the country’s renewable targets. We assume that the remaining 18% of generation is provided by existing gas generators with 15 GW capacity and the results are provided in Table 6.

**Table 6.** Generation and storage requirement to achieve 82% renewable penetration.

Region	Wind	Solar	Shallow		Medium		Deep		Cost
	(GW)	(GW)	(GW)	(GWh)	(GW)	(GWh)	(GW)	(GWh)	A$Bn
AEM	28.2	10.8	2.4	8.6	3.0	29	0.4	12.2	68.5

### Sensitivity analysis

To assess the robustness of our generation-storage requirements model, we conduct a range of sensitivity testing. Firstly, by investigating the assumption of single storage technology with associated cost and RTE to support the grid. Secondly, by varying the cost assumptions for power and storage system technologies. The costs of these technologies are important drivers in our model, and therefore, we evaluate the impact of different cost inputs replicating Monte Carlo simulation (MCS) for multiple random cost variations. Additionally, we conduct sensitivity for specific scenarios with given cost variation, which is outlined in the Supplementary material.

#### Technology sensitivity—single storage assumption

Three simulations are performed to investigate the AEM grid’s generation and storage requirements using only one type of storage technology, namely shallow, medium, and deep, with their respective costs and efficiencies as per the reference case. Table 7 shows the results, with the reference case having three storage types listed in absolute values, and the single technology results are presented as percentages relative to the reference values; the negative sign (−) indicates a percent decrease from the reference case and vice versa.

**Table 7.** Relative requirements of generation and storage with single storage technology assumption.

Scenario	Wind	Solar	Storage power	Storage energy	Cost	Curtailment
Reference (AEM)	38.2 GW	21 GW	18.2 GW	700 GWh	$129.1 billion	19.4 TWh
Shallow only	− 8%	+ 84%	+ 23%	− 74%	+ 21%	+ 156%
Medium only	− 12%	+ 66%	+ 11%	− 62%	+ 10%	+ 85%
Deep only	+ 9%	− 11%	− 2%	+ 12%	+ 4%	− 2%

The shallow or medium storage-only scenarios require higher solar and storage power capacities, with a decrease in wind and storage energy capacities. Utilizing shallow and medium storage leads to 8 and 13 h storage provision, respectively, with an overall cost increase of 21 and 10%; the study acknowledges that the cost assumptions for shallow storage were based on up to 4 h of storage, and using shallow storage for 8 h (as medium storage) would significantly increase the storage cost and overall costs. On the contrary, investing in deep storage greatly reduces renewable curtailment by increasing wind penetration and storage energy capacities, resulting in a larger reduction compared to shallow and medium storage. Notably, the storage capacity requirements are contingent upon the allowable curtailment, and the selection of storage has a defining impact on curtailment.

#### Cost sensitivity—random cost assumptions

**Fig. 7.**
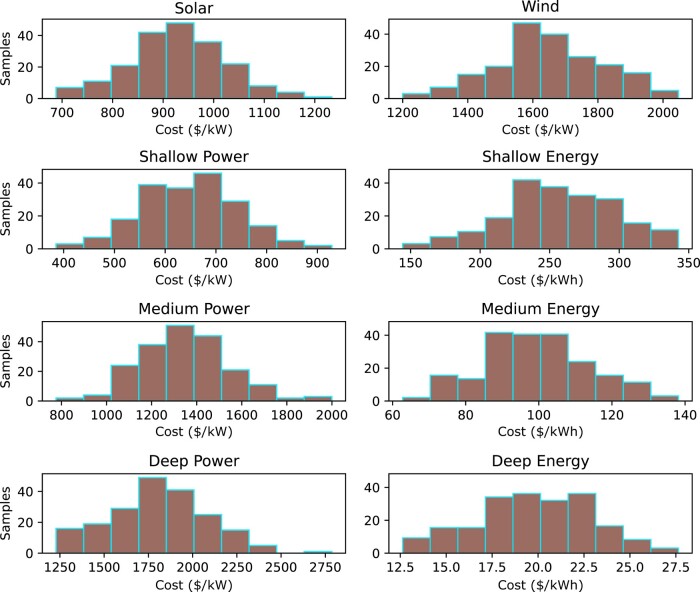
Random uniform distribution for technology costs as input to the optimization model to investigate optimal capacities and least cost solution.

**Fig. 8.**
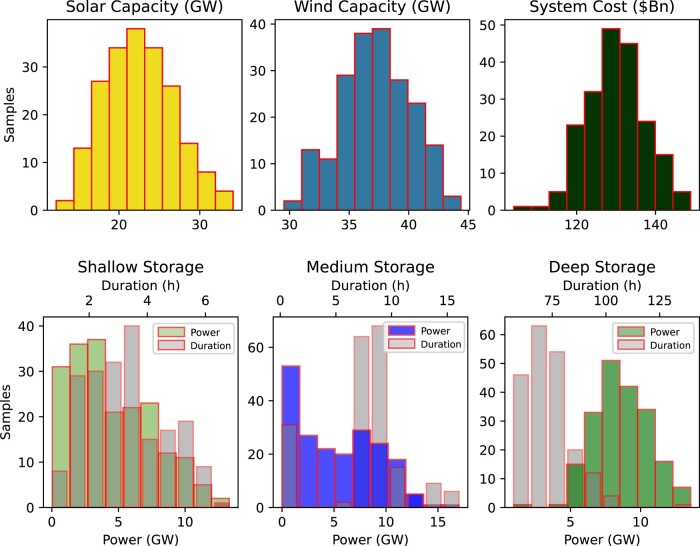
Generation and storage capacity distribution resulting from the MCS, with the power capacity (GW) and duration (h) of storage technologies overlaid. The resultant investment cost distribution is plotted as well.

**Fig. 9.**
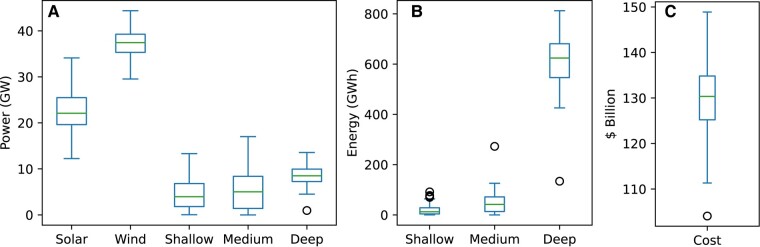
The a) power (GW), b) energy (GWh), and c) cost ($Bn) range as a result of MCS. The outliers in power and energy capacity for deep storage are due to the low-cost assumption of the medium storage energy component, resulting in an increased medium storage energy capacity. Additionally, the cost outlier is due to the low-cost assumption of wind technology.

Note that MCS is a statistical technique that uses random sampling to simulate a stochastic model and analyze uncertainty related to technology costs. The simulation is executed 200 times with random generation and storage costs, based on mean reference costs and SD of 10 and 15% for generation and storage costs, respectively. The random uniform distribution for the technology cost input to the model is shown in Fig. 7, while Figs. 8 and 9 display the results for generation and storage capacities, along with capital costs.

By utilizing robust optimization and sensitivity analysis, the varying costs of each technology are taken into account, leading to a range of potential capacities and optimal technology mixes. This approach provides a more comprehensive understanding of the overall limitations. Specifically, when considering a mix of solar and wind generation technologies, the capacity ranges from 12.2 to 34.1 GW for solar and 29.5 to 44 GW for wind; however, wind generation contributes half to one-third of energy demand across all scenarios. Storage capacities, including power and duration, are also influenced by the chosen generation mix and storage costs. Deep storage capacity is crucial in most scenarios, with power capacity ranging from 4.5 to 13.5 GW and duration from 56 to 100 h. However, an exception occurs with cheaper medium storage, as evident from the outliers in Fig. 9. Finally, the WA–SA interconnector’s optimum capacity ranges from 1.4 to 1.8 GW.

### Limitations and exclusions

The study’s limitations encompass excluding economics-driven changes to consumption patterns resulting from DSM and real-time market pricing. This intentional omission is due to the anticipated changes in generator merit-order and bidding systems in future electricity markets, primarily driven by renewable generators, as opposed to the current dispatch system. Similarly, grid-connected storage applications, including energy arbitrage, peaker plant, frequency regulation services, and the associated revenues, are not within the study’s purview. Our study primarily focuses on the technical requirements for the grid to operate fully with renewable energy, emphasizing storage for energy adequacy. Additionally, the increased storage integration is expected to reduce price volatility, resulting in lower profits from energy arbitrage (56). Consequently, future electricity market dynamics and storage revenues are deliberately excluded from the study’s considerations.

Furthermore, our investigation assumes a storage DOD of 100% and excludes self-discharge and storage degradation considerations to simplify the analysis. Storage technologies behave differently due to inherent operating principles, and accurately modeling the associated degradation is mathematically intensive and complex, diverting from our study’s objective. The DOD limitation and optimum operating envelope predominantly pertain to electrochemical batteries, which are subject to cyclic degradation and accelerated deterioration with deep discharges. In contrast, PHES, CAES, and PtH_2_tP do not have similar constraints. Nevertheless, the impact of these parameters on sizing requirements and overall investments is minimal and is well within the bounds of the sensitivity analysis.

The grid is expected to continuously experience increased loads from adopting electric vehicles, electric heating, and other forms of electrification. However, this study adopts a cautious approach and refrains from extrapolating the future demand based on any specific factor for three reasons. Firstly, the demand curve will likely change due to the introduction of smart grid appliances and demand shifting/management through real-time market pricing. Secondly, the self-consumption of rooftop-solar electricity is expected to increase, potentially altering the current “duck curve” behavior. Lastly, improvements in electrical efficiency are expected to offset up to 5–10% of additional demand.

## Discussion

The cost of storage energy and power capacity significantly affects the optimal generation mix (29) and overall cost. Increasing the amount of solar power in the grid will reduce the need for storage energy capacity (duration) but will lead to higher curtailment. On the other hand, incorporating more wind power can lower the storage power capacity and overall curtailment, requiring long-duration storage as demonstrated in the sensitivity analysis (refer to single storage assumption). The optimal low-cost solution, therefore, depends on the cost of integrated storage technologies. Despite the benefits of reduced cost, quicker installation, and a smaller physical footprint, solar technology has lower capacity factors than wind. Consequently, while solar generation is cheaper than wind, solar with storage will tend to be expensive in VRE-dominated grids, partly due to increased solar power curtailment resulting in increased cost of energy. Additionally, high penetration of solar power leads to lower capacity factors for transmission lines and interconnections. Our findings suggest that a generation mix comprising wind capacities ranging from 29.5 to 44 GW, supplying approximately 50 to 75% of the energy demand, is optimal to achieve a fully renewable electricity sector.

Storage provides backup to intermittent renewable sources, and by assuming storage efficiency and cost, we investigated the optimal storage mix. Although the study is limited by its reliance on consumption and generation data from a single year and multiyear analysis may suggest additional storage needs; nevertheless, we conduct sensitivity analysis to understand the bounds of capacity requirements. The sensitivity analysis reveals that the deep storage accounts for a significant portion of storage capacity, providing up to 3 days of backup and accounting for 46 and 91% (median values) of the overall storage power and energy capacity, respectively (refer to cost sensitivity). For PtH_2_tP technology using electrolyzer and fuel cell, the storage energy capacity corresponds to hydrogen storage of approximately 34 million tonnes per annum assuming 1 kg of hydrogen generating 19 kWh through a 60% efficient fuel cell, and roughly 40 plants similar to planned in SA Whyalla for power capacity (49).

The combined storage power capacities for shallow, medium, and deep storage range from 17.7 to 20.6 GW, with a median value of 18.4 GW, accounting for 54% of the peak demand in 2021. Similarly, according to sensitivity analysis, the combined energy capacities range from 400 to 840 GWh (Fig. 9), with a median value of 657 GWh, representing roughly 0.33% of the annual energy demand. Despite challenges in integrating deep storage technologies due to lower efficiencies, the future grid of RE100 will require significant storage capacities complemented by fast-acting short-term storage to mitigate potential energy shortfalls caused by periods of low wind and solar generation, which may extend across weeks or intraseasons, and to provide ancillary grid services.

Australia’s storage requirements for the fully renewable electricity sector are significantly higher than the existing storage capacities integrated with the grid, with 13 times more storage power capacity and over 40 times more storage energy capacity needed. Nonetheless, the storage energy capacity can potentially be reduced by over half by employing higher efficient storage systems, subject to cost reductions in shallow and medium storage technologies facilitated by technological learning and economies of scale (refer Table 7 and sensitivity analysis in Supplementary material). This may be possible with flow batteries capable of providing up to 8 h of storage (52). Additionally, much of the storage need arises as renewable penetration exceeds 90%. For instance, for an interim target of 82% by 2030, Australia will need to increase its storage power capacity by 4-fold and energy capacity by 3-fold and will need half of the investments required for a fully renewable grid. The dynamics of the least-cost optimal mix can be influenced by various factors, including the duration of storage, power, and energy costs associated with the specific technology and the efficiency of discharging (57).

Moreover, interconnectors between regions provide power transfer and desirably reduce dependence on medium or deep storage as transmission line losses are typically lower than storage losses, i.e. 5–10% compared to 15–40%, respectively. The interconnection of the NEM and the WEM not only enhances grid flexibility and helps to smooth out demand but also proves to be a cost-effective solution. The proposed interconnector contributes to a 14% reduction in storage power capacity. Furthermore, the AEM requires an investment of around A$131.4 billion, about A$5.3 billion less than isolated WEM and NEM grids, emphasizing the benefit of sharing generation and storage resources between regions. Improved regional connectivity generally leads to reduced regional generation and storage capacity requirements—an additional 22% generation capacity and 63% storage energy capacity is needed to cater to isolated grids scenario (refer Table 2). In terms of total investments, roughly 63% of the capital is allocated to the deployment of additional generation compared to one-third of the total investment on storage infrastructure; these findings align with the AEMO forecasts (39). Additionally, the interconnector provides opportunities to exploit all renewable resources across its transmission easement. It has also been observed that solar and wind generators have higher capacity factors in the WEM, and geo-shifting power to provide for the NEM will be possible with the NEM-WEM interconnector.

Due to the increased interest in renewable futures, related studies to investigate generation and storage requirements have been undertaken by organizations such as AEMO (39), CSIRO (58), and Net-Zero Australia (59). These studies focus on modeling scenarios for renewable transition and low-cost optimization, analyzing the electricity, transportation, and industry sectors to provide for at least double the current demand. While CSIRO’s study expands upon AEMO’s research and concentrates primarily on storage requirements, Net-Zero Australia’s study considers the potential for Australian exports and incorporates carbon capture, utilization, and storage (CCUS). In all studies, a small percentage of gas generation is presumed to provide firming in 2050. On the other hand, our study is an independent investigation that uses its own model, assumptions, and separate inputs. Despite this, our findings are consistent with those of other studies, which validate the overall corpus of work. Furthermore, our study examines the potential advantages of the WEM-NEM interconnector, which was not included in the above studies. All studies emphasize the importance of deep storage technologies and suggest their rapid development to reduce costs.

The Australian energy sector is swiftly embracing renewable technologies, prompting investments in both established (e.g. utility solar, onshore wind) and emerging solutions (e.g. offshore wind, electrification of transportation and industrial loads, community batteries, smart grids as virtual power plants). Challenges stem from the widespread adoption of rooftop solar, especially in the WEM, where grid isolation necessitates meticulous integration planning to manage distributed and largely uncontrolled generation, to ensure grid stability. Additionally, rooftop solar generation nears saturation, particularly in many local distribution networks, requiring additional investments for further connections. With solar generation’s capacity factors ranging from 25 to 30%, investments in transmission can be inefficient, leaving the grid underutilized at times. This can be countered with community batteries, which are charged by households having rooftop-solar only. Although community batteries offer potential for evening load smoothing and profitability for microgrid investors, yet fall short in meeting longer duration peak demands, particularly during summer and winter peaks.

Moreover, electric vehicles (EVs) present significant storage potential through V2G arrangements. However, their charging demands may compound grid congestion and peak demands, necessitating load-shifting strategies to optimize solar utilization and smoothening the demand curve. Nevertheless, V2G and VPP solutions can be aggregated to form a virtual energy storage system akin to conventional ESS, delivering comparable services.

Finally, note that deep storage requirements fluctuate from 2 to 4 days based on sensitivity analysis, and thus PtH_2_tP may potentially be a solution regardless of lower efficiencies. The interconnection between NEM and WEM may facilitate locating hydrogen generation at WA, with renewable electricity sourced from NEM; this can reduce the logistic costs compared with exporting hydrogen from western ports (48). The option to sell hydrogen to sectors outside of power generation provides an additional potential revenue stream for hydrogen technology systems.

## Conclusion

The transition to renewable technologies requires addressing the intermittency and inflexibility challenge associated with VRE sources. While several solutions exist, energy storage and the expansion of existing interconnectors will play a much larger role. Storage not only provides auxiliary services due to quick response (BESS) but is also essential for providing energy adequacy in the fully renewable grid. Our study proposes a generation-storage capacity optimization model for the Australian grid to investigate optimum least-cost solutions while proposing an interconnector between eastern and western grids. Furthermore, the sensitivity analysis gives a holistic view of the bounds for different technologies and anticipated investment costs for the transition.

The optimal mix of renewable generation and multitimescale storage is determined by minimizing the total capital cost of these components. The capacity requirements are sensitive to technology costs and storage efficiencies, and while renewable generation costs are relatively predictable, storage costs can be more uncertain. However, as storage technologies mature and costs decrease, higher levels of storage deployment may be possible than previously anticipated. Overgeneration of renewable energy is also necessary to reduce storage needs, but this can result in higher energy curtailment. To address this, additional regional interconnections can be established to reduce curtailment, while excess energy can be used to produce synthetic fuels and hydrogen for export and additional revenue (opportunity cost of curtailment). Moreover, by properly accounting for energy units across the NEM, the energy could be exported to the WEM to establish WA as the country’s hydrogen hub, utilizing the region’s existing mining and gas export infrastructure.

For a fully renewable NEM and WEM, the proposed interconnector with a capacity of 1.4–1.8 GW offers cost savings by reducing generation and storage requirements and alleviates curtailment and other storage-associated losses while offering greater grid flexibility. We represent storage with three different technologies based on their cost and RTE and determine the most optimal capacities. It is found that deep storage plays a crucial role in the RE100 scenario, accounting for 46 and 91% (median values) of the overall storage power and energy capacity, respectively. At the same time, the combined storage capacities account for 54 and 0.33% of the peak and annual energy demand, respectively. This storage mix is optimal at an overgeneration capacity of roughly 120%, with wind providing over 50% of total generated energy. Depending on their development, the optimal mix of generation and storage technologies along with an interconnector connecting NEM and WEM would require a conservative investment of approximately A$130–150 billion. This amount accounts for 8–10% of the country’s gross domestic product and can be amortized over a 10- to 15-year period to transition to a near-100% renewable grid.

## Supplementary Material

pgae127_Supplementary_Data

## Data Availability

The individual generation and regional demand data for 2021 can be downloaded from Shaikh (60).

## Preprints

This manuscript was posted on a preprint: http://dx.doi.org/10.2139/ssrn.4482528.
