# Supplementary material for: Robust capital cost optimization of generation and multitimescale storage requirements for a 100% renewable Australian electricity grid
Source: PNAS Nexus. 2024 Mar 25;3(4):pgae127. doi: 10.1093/pnasnexus/pgae127 (PMC10994200; doi:10.1093/pnasnexus/pgae127)
Supplement: pgae127_Supplementary_Data [file pgae127_supplementary_data.zip › PNASNEXUS-PNASNEXUS-2023-01008RR-s01.pdf]

Supplementary Material

Supplementary material for additional details on modelling can be accessed below.

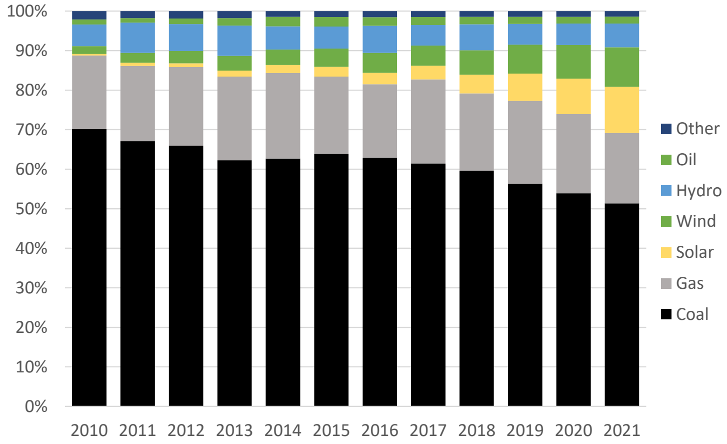

Fig. S-1. Fuel sources for electricity generation across ten years for Australia, based on data from BP statistics [2].

Methods and optimization model

Our study utilizes actual generation data of 100 generators (solar and wind) connected in the NEM and 16 generators connected in the WEM at 30-min time-series sampling intervals for the year 2021, with regional capacities provided in Table S-1. Additionally, we consider three storage options for each region: lithium-ion batteries (Li-ion), PHES, and power-to-gas-to-power (PtH_2tP), chosen based on their suitability for short-term (shallow), medium-term (medium), and long-term (deep) storage, respectively. In total, 18 storage systems are evaluated across six regions. To align with NEM's time zone for the model considering the interconnector between the two grids, we have adjusted the WEM's generation and demand output by 2 hrs.

Let the set $\mathcal{R}$ comprise the regions r of SA, VIC, NSW, QLD, TAS, and WA, and let $\mathcal{G}$ denote the set of utility-solar or wind generators. Then $\mathcal{G}(r)$ is the set of generators in region r. Similarly, let $\mathcal{H}$ represent the set of storage technologies h characterized by duration (hours), and let i be an interconnector in the set $\mathcal{I}$, where $\mathcal{I}(r)$ is the set of interconnectors that are incident on region r.

The optimization objective is to minimize the overall system costs, which comprise investment costs of generation (from solar and wind), various storage systems (based on duration), considering power (GW) and energy capacity (GWh) costs, and the cost of new interconnections; this optimization framework is adapted from [10], refer Fig. S-2. The objective function is given by:

$$\text{Min}\left(\sum_{r\in\mathcal{R}}\left(C_S P_S^r + C_W P_W^r + \sum_{h\in\mathcal{H}}\left(C_h^e E_h^r + C_h^p P_h^r\right)\right) + C_I\right), \tag{1}$$

where C_S and C_W denote the capital costs of solar and wind power plants (A\$/GW), respectively. C_h^e and C_h^p represent the investment costs of the storage energy capacity (A\$/GWh) and storage power capacity (A\$/GW), respectively. For each region $r \in \mathcal{R}$, P_S^r and P_W^r denote the nameplate capacities of additional generators, while E_h^r and P_h^r are the storage energy and power capacity (rated), respectively for each storage $h \in \mathcal{H}$ in each region $r \in \mathcal{R}$. Finally, C_I is the interconnector cost and comprises the cost of transmission lines.

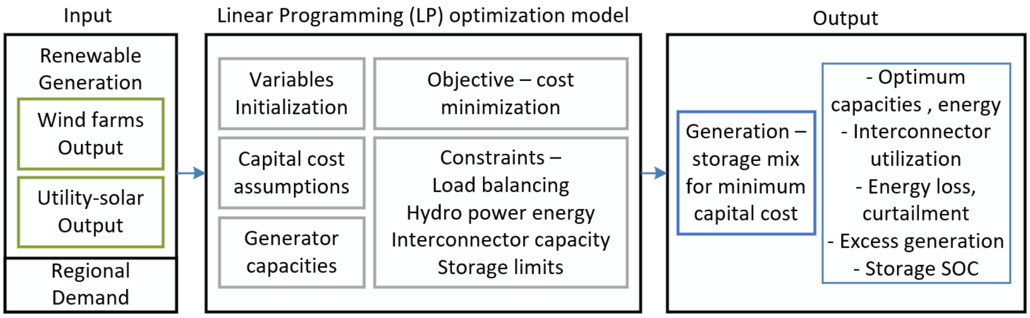

Fig. S-2. Linear Programming (LP) model with generation data from existing generators (total of 116 across Australia) to minimize the overall cost of generation and storage as adapted from [10].

Table S-1. Regional demand with generation data (time series) provided to the optimization model, taken from the number of existing generators, their combined capacities, and average capacity factor (CF) as of 2021.

Regions	Demand (TWh)	Utility-Solar			Onshore Wind		
		Number of plants	Capacities (MW)	CF (%)	Number of plants	Capacities (MW)	CF (%)
NSW	66.2	20	1,963	25	12	1,685	34
QLD	53.4	21	1,509	25	2	620	32
VIC	41.5	6	624	25	17	2,273	37
WA	17.6	4	152	26	12	992	39
SA	10.5	3	315	27	16	1,660	36
TAS	10.0	0	0	-	3	424	40
Total	199	54	4,563	25	62	7,654	35

The optimization model must satisfy several constraints, primarily (i) supply-demand balancing (Eq. 2) and (ii) storage state of charge (Eq. 6. The regional operational demand[1] at any time P^r_{Lt} is met with the regional generation. Subsequently, any deficit or excess is managed by importing or exporting electricity from inter-connectors P^r_{It}, charging or discharging the regional storage facilities, and regional curtailment P^r_{Xt}, then the power balance is depicted in Eq. 2,

$$P^r_{Gt} + P^r_{It} + \overbrace{P^{r-}_{ct}}^{P^{r-}_{ct} \leq 0} + \underbrace{P^{r+}_{dt}}_{P^{r+}_{dt} \geq 0} - \overbrace{P^r_{Xt}}^{P^r_{Xt} \geq 0} - P^r_{Lt} = 0 \quad \forall t \in \mathcal{T}, \quad \forall r \in \mathcal{R}, \tag{2}$$

where P^r_{Gt} is the cumulative power output from optimally scaled VRE generators and optimally dispatched hydro generators[2] within region r at time t, while P^{r-}_{ct} and P^{r+}_{dt} represent charging and discharging power at time t from all assumed storage technologies in region r. These parameters are represented as,

$$P^r_{Gt} = \sum_{g \in \mathcal{G}(r)} \beta_g P^0_{gt} + P^r_{Ht}, \tag{3}$$

$$P^{r-}_{ct} = \sum_{h \in \mathcal{H}(r)} P^{r-}_{cht}, \tag{4}$$

$$P^{r+}_{dt} = \sum_{h \in \mathcal{H}(r)} P^{r+}_{dht}. \tag{5}$$

Here P^0_{gt} is the actual power output of existing utility-solar and wind generators at time t, which is scaled by the factor β_g to optimally balance the power equation, and P^r_{Ht} is the optimally dispatched hydropower in region r at time t. Importantly, the scaling factor is constrained ($\beta_g \geq 1$) to ensure that at least the actual existing generator capacity is utilized in the model.

The operation of storage technology $h \in \mathcal{H}$ in region r requires that the energy in the storage at time t, i.e., its state of charge (SOC), be determined by the charge P^{r-}_{cht} and discharge P^{r+}_{dht} powers and the energy stored at the previous time-step as represented by Eq. 6,

$$E^r_{ht} = E^r_{h(t-1)} - \left(\eta_{ch} P^{r-}_{cht} + \frac{P^{r+}_{dht}}{\eta_{dh}} \right) \Delta t \quad \forall h \in \mathcal{H} \quad \forall t \in \mathcal{T} \quad \forall r \in \mathcal{R}, \tag{6}$$

where η_{ch} and η_{dh} are the charging and discharging efficiencies for each storage technology $h \in \mathcal{H}$, respectively, and Δt is the discretization interval.

In order to achieve a low-cost solution, we allow annual regional generation E^r_G to exceed the annual regional demand E^r_L by a factor α^r. Our model finds the optimum over-capacity factor (α^r_{opt}) for each region to achieve the least cost generation-storage mix while meeting the model constraints,

[1] The operational demand refers to the demand for electricity not met by rooftop-solar or home batteries, i.e., metered electricity.

[2] Existing regional hydro generators with a total of 7 GW capacity and an annual energy constraint of 13 TWh are assumed.

$$E_G^r = \alpha^r E_L^r \quad \forall r \in \mathcal{R}, \tag{7}$$

where,

$$E_G^r = \sum_{t\in\mathcal{T}} (P_{Gt}^r)\Delta t, \tag{8}$$

and,

$$E_L^r = (\sum_{t\in\mathcal{T}} P_{Lt}^r)\Delta t \quad \forall t \in \mathcal{T}, \quad \forall r \in \mathcal{R}. \tag{9}$$

The algorithm determines the required regional capacities for utiliy-solar P_S^r and wind P_W^r generators to determine the associated investments. Similarly, for storage, the energy capacity E_h^r is the difference of maximum and minimum energy levels ($E_h^{r,\max} - E_h^{r,\min}$), while the power capacity required for regional storage P_h^r is the maximum power discharged from storage h at any interval across the simulation year, i.e., $P_h^r = P_{dh}^{r,\max}$.

The choice between high-voltage transmission lines of alternating-current (HVAC) or direct-current (HVDC) depends on the infrastructure cost, as well as the cost and benefit of tapping into intermediate points along the route. Although HVAC is cheaper for shorter distances of 500-900 km and offers lower-cost connectivity, long-distance HVAC transmission requires intermediate switching stations and reactive power compensation, making HVDC costs comparable to HVAC. Bipolar HVDC transmission lines offer advantages such as a narrower right-of-way (ROW), smaller towers, and reduced line losses compared to similarly sized HVAC lines [9]. Therefore, we select HVDC technology for the proposed new interconnector connecting SA and WA, with a variable capacity P_I and a fixed length at 2400 km. The cost of a single bi-pole HVDC interconnector with a capacity of 1,200 MW (+/-500 kV) is estimated at A$2.58 billion [11], and this cost is used as a baseline for the determination of the optimal interconnector capacity in our study. We further assume that the line costs do not change significantly as the line capacity changes (within a reasonable range from the baseline).

This assumption probably underestimates the cost change from an increase in line capacity. On the other hand, we assume that converter stations and AC connection costs are proportional to the rating, which may overestimate the cost increase due to increased capacity. Since the errors in the two contributions to the cost are in opposite directions, there will be some cancellation of the errors inherent in these assumptions. Based on these cost assumptions, an interconnector with a one-gigawatt capacity and a length of 2400 km is estimated to cost approximately A$2.42 billion, while an interconnector with double capacity is estimated to cost approximately A$3.21 billion. It is noteworthy that having a cost assumption of $/kW-km would double the price of an interconnector with double capacity, and thus such an assumption is not used, as it would render the cost assumptions unreasonable. The cost of an interconnector C_I can be estimated using the equation provided,

$$C_I = L_I\,(C_l + C_e) + P_I\,(n_{\text{cs}}C_{\text{cs}} + n_c C_c)\,, \tag{10}$$

where, L_I and P_I represent the length and optimum capacity of the interconnector, respectively. The symbols of C_l, C_e, C_{cs}, and C_c represent the components costs of the interconnector, that is, the transmission line, easements, converter station, and AC connection, respectively; while n_{cs} and n_c indicate the number of converter stations and connections (a pair of each is assumed for our model). Figure **??** shows the schematic diagram of the interconnected NEM and WEM with a hypothetical transmission line across WA and SA, along with existing interconnector power capacities, with a transmission power loss of 5% assumed for any energy transfer. The distribution networks within each NEM and WEM region are not modelled, and therefore only utility-scale generation, storage, and inter-regional interconnectors are considered.

Table S-2. Existing and expected storage capacities for Australia as of 2022.

Projects	Storage Technology					
	BESS			PHES		
	Total	Power (GW)	Energy (GWh)	Total	Power (GW)	Energy (GWh)
Existing	7	0.58	0.802	3	0.53	12.2
Committed	9	0.61	0.83	2	0.25	2
Announced	43	10.6	24	5	3.3	23
Anticipated	7	1.29	3.8	-	-	-

Other than Snowy 2.0 PHES (2 GW / 350 GWh)

Techno-economic assumptions

The total installed costs for utility-solar PV and onshore wind power plants have been reduced to a global average of A$1,177 per kW and A$1,807 per kW, respectively, as of 2020 [7]. Furthermore, the storage cost comprises its power capacity ($/kW) and energy capacity ($/kWh) costs. On a $/kWh basis, deep storage has a lower capital cost, and on a $/kW basis, shallow storage has a lower capital cost [4]. The storage cost for deep storage tends to be the cheapest and tends to decrease with increasing storage duration, whereas storage energy capacity is the most costly for shallow storage. Therefore, batteries tend to be more competitive in short-duration storage applications, while PHES, CAES, and PtH_2tP are more competitive in high-duration applications.

Because the cost breakdown changes significantly with duration, storage costs for shallow, medium, and deep storage with the duration of 4, 12, and 24 hrs, respectively, are considered. The batteries with a duration of up to 4 hrs have a cost range of 400–800 $/kWh; however, the prices are expected to decline with a projected cost of 264 $/kWh by 2030 and 200 $/kWh by 2050 [4]. The economic assumptions for the year 2030 in Australian dollars (1 AUD = 0.75 USD) are listed in Tables S-3, S-4, and S-5; note that Table S-3 lists generation costs adapted from CSIRO GenCost [5] and IRENA [7, 6], Table S-4 provides the storage system parameters derived from NREL [4], McKinsey [8], and CSIRO National Hydrogen Roadmap [3], and Table S-5 has HVDC interconnection parameters sourced from Bogdanov *et al.* [1].

Table S-3. Cost of solar and wind technologies for the year 2030.

Technology	Unit	Solar	Wind
Capex	$/kW	950	1650

Table S-4. Shallow, medium, and deep storage cost and lifetime parameters.

Parameters	Unit	Shallow (4 hrs)	Medium (12 hrs)	Deep (24 hrs)
Charger/discharger	$/kW	650	1350	1800
Storage reservoir	$/kWh	260	110	20
Efficiency η_c/η_d	%	95/95 (RTE=90)	90/90 (RTE=80)	80/60 (RTE=48)

Table S-5. Cost parameters for HVDC transmission.

Parameter	Unit	Value
Overhead cable	$Mn/km	0.625
Easements	$Mn/km	0.054
Converter station	$/kW	375
AC connection	$/kW	20.8

Optimization model's methodology and results

Table S-6. Electricity generation and storage for different scenarios and regions.

Regions	α_{opt}	Wind		Solar		Shallow		Medium		Deep		Cost (Billion)		
		P (GW)	E (TWh)	P (GW)	E (TWh)	P (GW)	E (GWh)	P (GW)	E (GWh)	P (GW)	E (GWh)	Generation	Storage	Total
Copper-Plate (CP)														
AEM	1.12	32.81	147.26	21.38	62.80	4.43	10.78	2.05	11.48	10.06	308.45	74.45	33.9	108.32
Isolated Grids (IG)														
NSW	1.27	16.99	58.60	8.43	23.42	2.51	7.02	1.29	11.54	3.69	172.41	36.05	16.4	52.49
QLD	1.30	8.27	25.82	16.07	42.80	0.07	0.27	5.06	43.58	2.89	339.32	28.91	23.3	52.20
VIC	1.28	6.97	38.88	4.59	11.77	1.17	2.94	0.79	4.67	3.20	284.56	15.86	14.5	30.36
WA	1.46	3.78	17.45	3.28	8.32	1.05	2.49	0.45	4.17	1.54	123.81	9.35	7.6	16.97
SA	1.71	2.22	13.40	1.56	4.65	0.60	1.74	0.27	2.03	1.35	108.90	5.15	6.0	11.16
TAS	1.0	0.2	2.1	0.0	0.0	0.0	0.0	0.0	0.0	0.0	0.0	0.3	0.0	0.3
AEM	1.3	38.4	156.2	33.9	91.0	5.4	14.5	7.9	66.0	12.7	1029.0	95.6	67.9	163.4
Bussiness as Usual (BAU)														
NSW	1.16	17.06	57.0	5.84	17.74	1.87	5.8	0.60	4.72	2.82	110.9	33.7	11.3	45.0
QLD	1.23	9.02	27.4	13.82	37.54	0.64	1.8	4.13	35.14	2.64	284.4	28.0	20.4	48.4
VIC	1.14	5.57	41.7	0.92	3.42	1.15	2.2	0.00	0.02	2.61	130.9	10.1	8.6	18.7
WA	1.46	3.78	17.5	3.28	8.32	1.05	2.5	0.45	4.17	1.54	123.8	9.4	7.6	17.0
SA	1.64	2.57	15.0	0.62	2.29	0.76	2.0	0.00	0.00	0.58	20.7	4.8	2.5	7.3
TAS	1.00	0.18	2.1	0.00	0.00	0.00	0.0	0.00	0.00	0.00	0.0	0.3	0.0	0.3
AEM	1.22	38.18	160.7	24.47	69.32	5.47	14.3	5.18	44.05	10.19	670.8	86.3	50.4	136.7
Australian Electricity Market (AEM)														
NSW	1.14	17.03	57.8	5.01	15.43	2.07	7.1	0.08	0.62	2.40	108.0	32.9	9.8	42.7
QLD	1.24	9.07	27.7	13.90	37.78	0.80	2.3	3.85	33.09	2.73	308.2	28.2	20.7	48.9
VIC	1.08	5.53	40.8	0.12	1.62	0.60	1.6	0.18	1.37	2.28	119.0	9.2	7.7	16.9
WA	1.47	4.17	20.6	2.01	5.25	0.36	1.0	0.30	2.20	0.86	52.9	8.8	3.7	12.5
SA	1.35	2.18	13.5	0.00	0.75	0.32	1.2	0.29	1.92	1.02	59.2	3.6	4.1	7.7
TAS	1.00	0.18	2.1	0.00	0.00	0.00	0.00	0.00	0.00	0.00	0.0	0.3	0.0	0.3
AEM	1.19	38.17	162.4	21.04	60.83	4.16	13.2	4.69	39.20	9.30	647.4	83.0	46.1	129.1

State of charge (SOC)

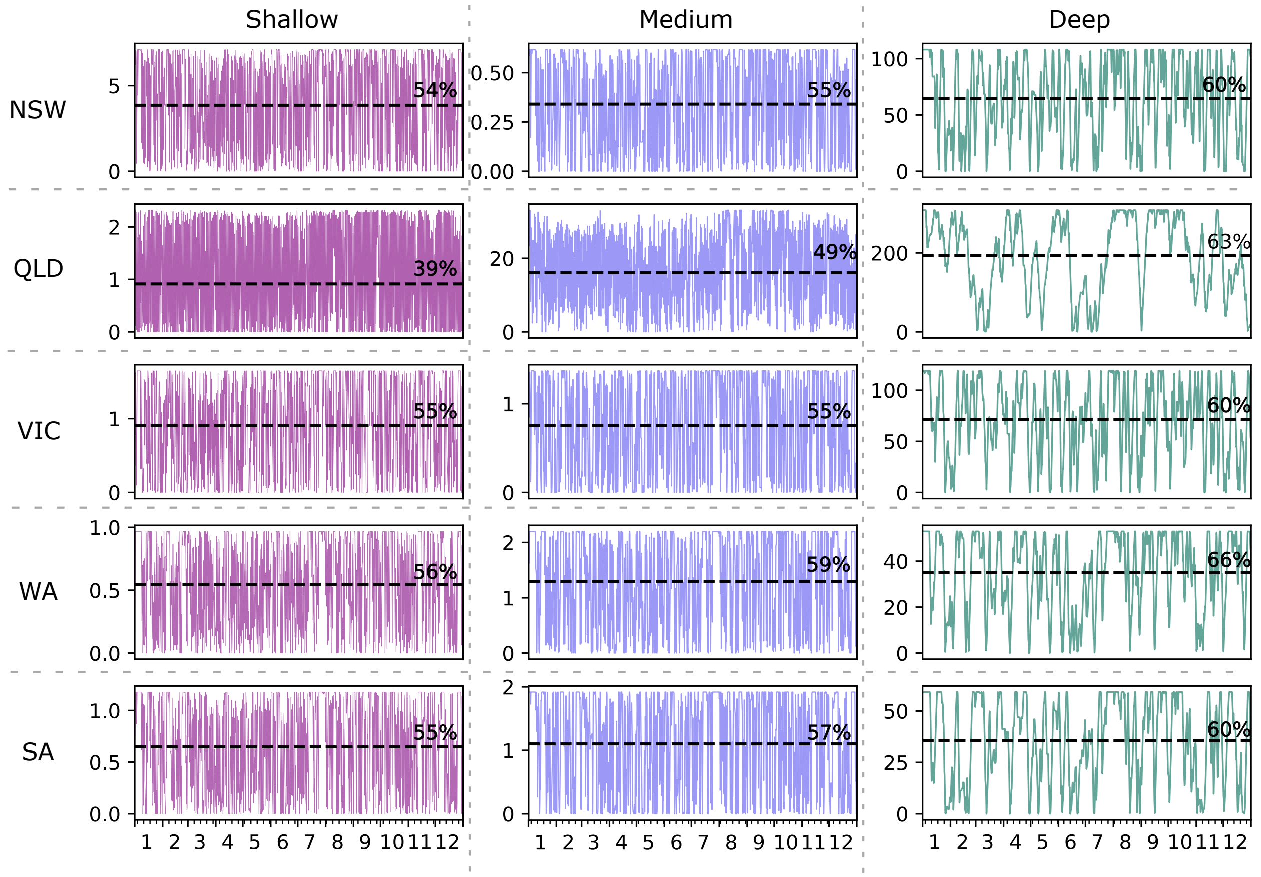

Fig. S-3. The response for shallow, medium, and deep storage for one year of simulation, with numbers on the horizontal axis indicating months, while the vertical axis indicates the storage capacity in GWh. The horizontal line indicates the average SOC of the storage annotated in per cent.

Cost sensitivity—Specific scenario assumptions

Table S-7 summarizes the percentage changes under the high and low-cost scenarios for each technology, where the reference cost is provided earlier in Table S-3 and Table S-4. We assume a cost variation range of 10–30% in consideration of plausible future scenarios. For the high scenario, we assume a 30% increase in cost for all technologies except solar due to ongoing developments and greater cost reduction potential. Similarly, shallow and deep storage may be developed to reduce the cost by 20% than the reference cost, whereas for medium storage, we take only 10% of the cost reduction.

Finally, we assume that solar generation costs will decline more competitively than wind [5], especially through rooftop implementation, and therefore solar is expected to have a 30% cost reduction in the low scenario, compared to 10% for wind. Sensitivity analysis is performed for the nine scenarios listed in Table S-8, and the results are plotted in Fig. S-4. The generation (GEN) and storage (STR) are tested individually for high and low costs, followed by specific technology changes. For instance, the scenario labelled W_H_&_S_L assumes high wind costs and low solar costs.

Table S-7. Sensitivity analysis basis for increase (high) and decrease (low) in costs of technologies.

Cost Sensitivity	Wind (%)	Solar (%)	Shallow (%)	Medium (%)	Deep (%)
High	+30	+10	+30	+30	+30
Low	−10	−30	−20	−10	−20

Table S-8. Specific sensitivity scenarios with identifiers and definitions.

No.	Scenarios	Identifiers	Definition
1	High storage cost	STR_H	All storage investment costs (power and energy) are increased and decreased, respectively, while keeping baseline generation costs.
2	Low storage cost	STR_L	
3	High generation cost	GEN_H	Keeping baseline storage costs, generation (wind and solar) investment costs are increased and decreased, respectively.
4	Low generation cost	GEN_L	
5	High wind and Low solar	W_H_&_S_L	Wind investment costs are increased, solar investment costs are decreased, and storage costs are kept at baseline.
6	Low solar and shallow storage cost	S_L_&_SH_L	Solar and shallow storage investment costs are decreased, while wind, medium, and deep storage costs are kept at baseline. These assumptions are to test a scenario where solar and batteries are deployed at a larger scale.
7	High deep storage cost	DP_H	Deep storage costs increase while keeping other costs at baseline. The deep storage (enabler of PtH_2tP) is far less developed than medium (PHES) storage.
8	Low deep storage cost	DP_L	Deep storage costs decrease
9	Low generation and High storage cost	GEN_L_&_STR_H	Generation costs are reduced, while storage costs are increased. This assumption is to verify the scenario where generation technologies are developed only.

The results indicate a general increase in solar capacity and a decrease in deep storage energy capacity across most scenarios, except when deep storage costs are reduced. A reduction of 30% in the specific investment costs of shallow storage causes a significant shift towards the use of shallow storage, as indicated by an increase in the EP_c ratio up to 6 hrs, which points to the development of shallow storage as a medium storage solution.

Furthermore, our findings indicate that the combined power capacities vary across different cost scenarios. The scenario with high wind and low solar costs has the highest capacity at 90 GW, while the scenario with low storage costs exhibits the lowest capacity at 76.5 GW. In terms of investment costs, the scenario with high generation costs requires the highest investment of A$149 billion, whereas the scenario with low generation costs incurs the lowest cost of A$116 billion. Additionally, the scenario with low generation and high storage costs results in the lowest accumulative storage energy capacity of 544 GWh, compared to 786 GWh in the scenario with low deep storage costs. These findings emphasize the significance of technology cost assumptions in making informed investment decisions for power and storage systems.

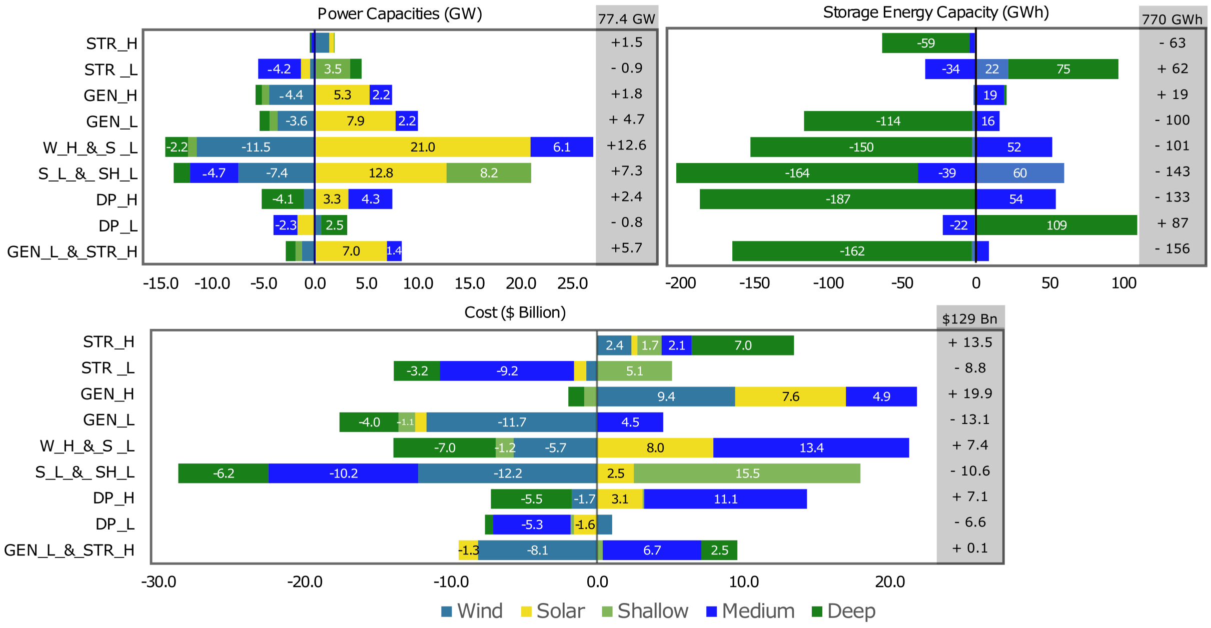

Fig. S-4. Sensitivity analysis due to changes in technology cost. The high (H) and low (L) cost assumptions as of percentage in Table S-8 for nine different scenarios against the reference cost (point 0). The change in required power capacity (top left), storage capacity (top right), and cost (bottom) with respect to the corresponding baseline quantities are shown. The baseline value for each variable is indicated in the top right corner of each plot, and the total change in each quantity from the reference scenario is shown.

Generators with the Identification number

Table S-9. Information of the generators used for the modelling and time-series input.

DUIDs.	Capacity (MW)	CF
South Australia		
BNGSF1	110	23%
BNGSF2	110	29%
TBSF1	95	21%
BLUFF1	53	31%
HALLWF1	95	39%
HALLWF2	71	37%
HDWF1	100	40%
HDWF2	100	37%
HDWF3	109	37%
LKBONNY2	159	29%
NBHWF1	132	38%
CLEMGPWF	57	35%
LGAPWF1	126	40%
LKBONNY3	39	29%
SNOWNTH1	144	35%
SNOWSTH1	126	39%
SNOWTWN1	99	40%
WATERLWF	131	31%
WGWF1	119	38%
Victoria		
BANN1	88	26%
GANNSF1	50	27%
KARSF1	90	26%
NUMURSF1	108	25%
WEMENSF1	88	25%
KIAMSF1	200	22%
ARWF1	241	28%
BALDHWF1	107	38%
MACARTH1	420	24%
OAKLAND1	25	86%
BULGANA1	204	33%
CHYTWF1	58	37%
DUNDWF1	168	38%
DUNDWF2	46	34%
DUNDWF3	122	38%
CROWLWF1	80	39%
KIATAWF1	31	49%
MERCER01	131	31%
MTGELWF1	132	26%
MUWAWF1	226	42%
SALTCRK1	54	36%
YENDWF1	144	28%
ELAINWF1	84	31%

DUIDs.	Capacity (MW)	CF
New South Wales		
BERYLSF1	87	21%
BROKENH1	53	26%
COLEASF1	150	26%
FINLYSF1	133	21%
NEVERSF1	105	27%
NYNGAN1	102	25%
BOMENSF1	102	21%
GOONSF1	70	27%
GULLRSF1	10	22%
LIMOSF21	29	29%
MANSLR1	47	23%
MOREESF1	56	24%
PARSF1	51	28%
WRSF1	20	22%
LIMOSF11	221	26%
SUNRSF1	200	23%
WELLSF1	170	18%
JEMALNG1	50	24%
DARLSF1	275	23%
MOLNGSF1	32	22%
BODWF1	113	34%
CROOKWF2	94	36%
GULLRWF1	166	33%
GUNNING1	47	39%
SAPHWF1	270	33%
STWF1	198	43%
TARALGA1	107	32%
WOODLWN1	48	34%
WRWF1	172	33%
BOCORWF1	113	34%
COLWF01	219	27%
CRURWF1	138	32%
Tasmania		
GRANWF1	112	42%
MUSSELR1	168	39%
CTHLWF1	144	39%

DUIDs.	Capacity (MW)	CF
Queensland		
CHILDSF1	56	23%
CLARESF1	100	25%
CLERMSF1	75	26%
CSPVPS1	43	22%
DAYDSF1	150	24%
DDSF1	110	20%
OAKEY1SF	25	25%
OAKEY2SF	55	23%
RUGBYR1	65	29%
MARYRSF1	27	26%
EMERASF1	72	28%
HAMISF1	56	26%
HAUGHT11	100	26%
HAYMSF1	50	24%
KSP1	49	29%
LILYSF1	100	28%
RRSF1	116	26%
SRSF1	75	24%
WHITSF1	56	25%
YARANSF1	103	26%
MIDDLSF1	26	27%
COOPGWF1	440	34%
MEWF1	180	29%
Western Australia		
ALINTA_WWF	86	45%
BADGINGARRA_WF1	132	49%
YANDIN_WF1	210	42%
WARRADARGE_WF1	178	42%
INVESTEC_COLLGAR_WF1	206	36%
EDWFMAN_WF1	83	39%
MWF_MUMBIDA_WF1	57	42%
MERSOLAR_PV1	101	30%
GREENOUGH_RIVER_PV1	40	28%
DCWL_DENMARK_WF1	1.44	44%
SKYFRM_MTBARKER_WF1	2.6	31%
ALBANY_WF1	21.6	30%
GRASMERE_WF1	13.8	35%
BREMER_BAY_WF1	0.61	35%
AMBRISOLAR_PV1	0.96	25%
NORTHAM_SF_PV1	9.9	23%

References

1. Dmitrii Bogdanov and Christian Breyer. North-East Asian Super Grid for 100% renewable energy supply: Optimal mix of energy technologies for electricity, gas and heat supply options. *Energy Conversion and Management*, 112:176–190, 2016.
2. BP. Statistical review of world energy. Technical report. Accessed on: 25/10/2022.
3. Bruce S, Temminghoff M, Hayward J, Schmidt E, Munnings C, Palfreyman D, and Hartley P. Pathways to an economically sustainable hydrogen industry in Australia National Hydrogen Roadmap. 2018.
4. Wesley Cole, A Will Frazier, and Chad Augustine. Cost projections for utility-scale battery storage: 2021 update. Technical report, National Renewable Energy Lab.(NREL), Golden, CO (United States), 2021.
5. Paul Graham, Jenny Hayward, James Foster, and Lisa Havas. GenCost 2020-21: Final report. *CSIRO*, 2021.
6. IRENA. *Green Hydrogen Cost Reduction: Scaling up Electrolysers to Meet the 1.5C Climate Goal, International Renewable Energy Agency, Abu Dhabi.* 2020.
7. IRENA. Renewable Power Generation Costs in 2019. Technical report, 2020.
8. McKinsey. Net-zero power long duration energy storage for a renewable grid, 2021. Accessed on: 05/01/2022.
9. Ahmad Shah Ayobe and Surbhi Gupta. Comparative investigation on HVDC and HVAC for bulk power delivery. *Materials Today: Proceedings*, 48:958–964, 2021.
10. Raheel A. Shaikh, David J. Vowles, Andrew Allison, and Derek Abbott. Evaluation of Australia's generation-storage requirements in a fully renewable grid with intermittent and flexible generation. *IEEE Access*, 2023. `http://dx.doi.org/10.1109/ACCESS.2023.3286037`.
11. Prof Simon and Bartlett Am. Investigation of HVDC Trans-Australian interconnections. Technical report, 2019.
